# Validation of Rapid Antimicrobial Susceptibility Testing directly from blood cultures using WASPLab^®^, including Colibrí^™^ and Radian^®^ in-Line Carousel

**DOI:** 10.1007/s10096-022-04421-8

**Published:** 2022-02-25

**Authors:** Pauline Hilda Herroelen, Robbe Heestermans, Kristof Emmerechts, Kristof Vandoorslaer, Ingrid Wybo, Denis Piérard, Astrid Muyldermans

**Affiliations:** Department of Microbiology and Infection Control, Vrije Universiteit Brussel (VUB), Universitair Ziekenhuis Brussel (UZ Brussel), Laarbeeklaan 101, B-1090 Brussels, Belgium

**Keywords:** Rapid AST, Direct AST, WASPLab^®^, Colibrí™, Radian^®^ in-Line Carousel, Blood culture

## Abstract

With the increase in antimicrobial resistance, fast reporting of antimicrobial susceptibility testing (AST) results is becoming increasingly important. EUCAST developed a method for rapid AST (RAST) directly from the broth of positive blood cultures (BC). Inhibition zones are read after 4, 6, and 8 h, with specific breakpoints per time point. We evaluated the RAST method based on EUCAST disk diffusion methodology with inoculation of BC broth using WASPLab^®^ (inclusive Colibrí^™^ and Radian^®^). Forty-nine non-duplicate strains were tested: *Escherichia coli n* = 17, *Klebsiella pneumoniae n* = 7, *Pseudomonas aeruginosa n* = 4, *Acinetobacter baumannii n* = 2, *Staphylococcus aureus n* = 10, *Enterococcus faecalis n* = 6, and *Enterococcus faecium n* = 3. Results were compared to direct AST and standardized AST. Good categorical agreement was obtained at all time points for all groups, except *P. aeruginosa*. RAST cut-offs for extended-spectrum β-lactamase (ESBL)-producing *Enterobacterales* enabled the detection of all included ESBL isolates (*n* = 5) at all time points, except for 1 *E. coli* ESBL after 4 h. RAST cut-offs for carbapenemase-producing *Enterobacterales* enabled the detection of only one carbapenemase after 6 h. However, all carbapenemases (*n* = 3) were correctly detected after 8 h. Two methicillin-resistant *S. aureus* were included; both were correctly categorized as cefoxitin-resistant at 6 and 8 h. At 4 h, there was insufficient growth for inhibition zone interpretation. EUCAST RAST is a fast supplementary tool for direct AST of positive BC. WASPLab^®^ provides a significant advantage as pictures are made automatically implicating that we are not strictly bound to the time points for inhibition zone interpretation.

## Introduction

Identification and antimicrobial susceptibility testing (AST) of pathogens are the most important tasks of a clinical microbiology laboratory. Especially, pathogens growing in blood cultures (BC) are of paramount importance. Bacteremia is a condition that can lead to severe complications, such as sepsis or septic shock, with high mortality rates [1–3]. It has been shown that early initiation of effective antibiotic therapy is an important prognostic factor in the management of bacteremia [4]. The management of sepsis has to balance between the most effective and the most responsible prescribing [5]. With the increase in antimicrobial resistance [6, 7], fast reporting of AST results is becoming increasingly important. Disk diffusion is one of the most frequently used methods for AST. The standardized European Committee on Antimicrobial Susceptibility Testing (EUCAST) method was developed for 16–20 h of incubation [8]. The process of identification and AST from clinical samples can thus take between 24 and 72 h [9]. Direct AST (DAST) by disk diffusion is a method for AST directly from the broth of a positive BC [10, 11]. Considering that no primary isolation of pathogens is necessary, this method speeds up the AST process by 1 day [10, 11]. Similarly as with standardized AST, inhibition zones are read after 16–20 h of incubation. However, this method is not standardized nor recommended by EUCAST. Recently, EUCAST has developed a method for rapid AST (RAST) directly from the broth of a positive BC [12]. Inhibition zones are read after 4, 6, and 8 h, with specific breakpoints per time point [13]. This allows rapid initiation of targeted antimicrobial therapy, reducing mortality and shortening the length of hospitalization [7, 9, 14].

Over the last decade, laboratory automation has improved productivity, traceability, and quality in clinical laboratories. For microbiological analyses, WASPLab^®^ (Copan, Brescia, Italy) was developed, which automates inoculation of specimens, incubation of agar media, and image analysis of bacterial cultures [15, 16]. Additionally, Colibrí™ (Copan) and Radian^®^ in-Line Carousel (Copan) were recently launched. Colibrí™ is an automated system that picks colonies selected with WASPLab^®^ and prepares them for identification through MALDI-TOF and processes 0.5 McFarland (McF) suspensions for AST [17]. Radian^®^ in-Line Carousel is a fully automated WASPLab^®^ module for disk diffusion AST and interpretation. It can manage up to 50 different antibiotic cartridges for flexible disk disposition on the plate [18].

The RAST method is time-consuming, and inhibition zones should be read strictly within 5 min of the stated reading time. To address these shortcomings, we evaluated the automation of the EUCAST RAST method for positive BC bottles using WASPLab^®^, inclusive Colibrí™, and Radian^®^ in-Line Carousel. Results were compared to direct AST (DAST) and standardized AST.

## Materials and methods

### Strains

This study was approved by the Ethics Committee of UZ Brussel (BUN 1432021000506). We tested 49 strains from clinical samples (*n* = 27) and collections of clinical isolates, conserved at − 80 °C (*n* = 22): *Escherichia coli n* = 17, *Klebsiella pneumoniae n* = 7, *Pseudomonas aeruginosa n* = 4, *Acinetobacter baumannii n* = 2, *Staphylococcus aureus n* = 10, *Enterococcus faecalis n* = 6, and *Enterococcus faecium n* = 3. We included five extended-spectrum β-lactamase (ESBL)-producing *Enterobacterales* (*E. coli n* = 3, *K. pneumoniae n* = 2), three carbapenemase-producing *Enterobacterales* (CPE) (OXA-48-producing *E. coli n* = 1, OXA-48-producing *K. pneumoniae n* = 1, NDM-1-producing *K. pneumoniae n* = 1), and two methicillin-resistant *S. aureus* (MRSA).

The clinical samples were prospectively analyzed positive BC (BACT/ALERT FA and FN Plus, bioMérieux, Marcy-l'Étoile, France), that were shown to contain *E. coli*, *K. pneumoniae*, *P. aeruginosa*, *A. baumannii*, *S. aureus*, *E. faecalis*, and *E. faecium*. Contaminated BC, BC containing other bacterial species, or polymicrobial BC were excluded. The BC were taken from hospitalized patients at University Hospital Brussels, between April 2021 and August 2021. Only one positive BC bottle per patient was processed. For the conserved isolates, we spiked BC bottles with 1 mL bacterial suspension of 100–200 colony-forming units (CFU)/mL and 5 mL sterile defibrinated horse blood (bioTRADING Benelux BV., Mijdrecht, the Netherlands). The BC bottles were incubated in the BACT/ALERT VIRTUO (bioMérieux). Identification of the included bacterial species was performed by matrix-assisted laser desorption ionization–time of flight mass spectrometry (MALDI-TOF MS), using the MALDI Biotyper^®^ (Bruker, Bremen, Germany), following manufacturers’ instruction.

### AST methodology

Positive BC were processed in parallel in three ways: (i) manually (DAST), (ii) using WASPLab^®^ and Radian^®^ in-Line Carousel (RAST), and (iii) using WASPLab^®^, Colibrí™, and Radian^®^ in-Line Carousel (standardized AST).

#### Direct AST

DAST was performed using disk diffusion methodology. This method represents the routine AST method for BC in our laboratory. Four drops of positive BC broth were added to 1 mL of 0.9% saline solution. Four drops of this inoculum were applied to a 120 mm square Mueller Hinton (MH) agar (Axonlab, Hengersberg, Germany), and 16 antibiotic disks (i2a, Montpellier, France) were added. The MH agars were incubated for 18 ± 2 h in a non-CO_2_ incubator (Thermo Fisher Scientific, Waltham, USA). Inhibition zones were read using SIRScan 2000 (i2a) [19].

#### Rapid AST

The inoculum for RAST was prepared following manufacturers’ instruction, starting from a dilution of 1 mL of positive BC broth with 2 mL of WASP PBS solution (Copan) [20]. We used 60 µL (2 × 30 µL loop/spreader) of this suspension as inoculum, which was applied to two circular 90 mm MH agars (Thermo Fisher Scientific) by WASP^®^. Six antibiotic disks (Thermo Fisher Scientific) were added to each agar, using the Radian^®^ in-Line Carousel. Agars were incubated in a WASPLab^®^ incubator, and inhibition zones were read after 4, 6, and 8 h, using WASPLab^®^ Webapp. EUCAST breakpoints for short incubation are reported as susceptible (S), area of technical uncertainty (ATU), or resistant (R) [13].

#### Standardized AST

Standardized AST was performed according to EUCAST standardized disk diffusion methodology adapted following the manufacturers’ instructions for inoculation by WASP^®^ [20]. One µL of diluted BC broth was inoculated on a non-selective blood agar (Thermo Fisher Scientific) by WASP^®^. This agar was incubated for 16 h in a WASPLab^®^ CO_2_ incubator. Subsequently, a 0.5 McF suspension was made from grown colonies by Colibrí™ as well as a 1/3 dilution of this suspension in 0.9% saline. This diluted suspension was used as inoculum for AST, being processed by the WASPLab^®^ system as described for RAST but with reading of inhibition zones after 18 ± 2 h [19].

### Quality control

BC bottles were spiked with 1 mL bacterial suspension of 100–200 CFU/mL of *E. coli* ATCC^®^ 25922™ or *S. aureus* ATCC®^®^ 29213™ and 5 mL of sterile defibrinated horse blood. The inoculated bottles were incubated in the BACT/ALERT VIRTUO and processed according to RAST and standardized AST methodology following a positive signal. We tested 24 ATCC^®^ 25922™ and 24 ATCC^®^ 29213™ over 5 days.

### Antimicrobials

To target both Gram-positive (GP) and Gram-negative (GN) bacteria, six antibiotic disks (Thermo Fisher Scientific) were added to each MH agar. For GP coverage, we tested ampicillin, cefoxitin, cefepime, clindamycin, erythromycin, and vancomycin. For GN coverage, we tested piperacillin-tazobactam, meropenem, ceftazidime, ciprofloxacin, amikacin, and trimethoprim-sulfamethoxazole. However, only antimicrobials with EUCAST breakpoints for short incubation (v 3.0) were included for analysis [13]. Screening for ESBL-producing *Enterobacterales*, carabapenemases, and MRSA was performed using ceftazidime, meropenem, and cefoxitin, respectively.

### Statistical analysis

Statistical analyses were performed using Microsoft Excel (version 2016, USA) and Medcalc (version 12.2.1, Belgium). Categorical agreement (CA), percentages of very major errors (VME), major errors (ME), and minor errors (mE) were calculated, based on standardized AST as reference method [21]. χ2 test was used for comparing proportions of categorical variables. Differences were considered statistically significant if *P* < 0.05.

## Results

### *Enterobacterales*

Results of RAST and DAST for Gram-negative bacteria are displayed in Table 1. We obtained good overall CA for every time point. The highest CA was obtained when interpreting inhibition zones after 8 h of incubation. However, no significant difference was observed between different time points or compared to DAST. We observed eight discordant results after 4 h of incubation (2 VME, 1 ME, 5 mE), six after 6 h (1 VME, 1 ME, 4 mE), and five after 8 h (1 VME, 4 mE). On the other hand, we observed 13 discordant results when using DAST (5 VME, 8 mE). RAST cut-offs for ESBL enabled the detection of all five ESBL isolates, at all time points, except for 1 *E. coli* ESBL when read after 4 h of incubation. RAST cut-offs for CPE enabled the detection of only one carbapenemase after 6 h of incubation; however, all carbapenemases (*n* = 3) were correctly detected after 8 h.

**Table 1:** Results of rapid AST and direct AST for Gram-negative bacteria, as compared to the standardized AST

		Rapid AST, 4 h						Rapid AST, 6 h						Rapid AST, 8 h						Direct AST			
		UR, *n* (%)	ATU, *n* (%)	CA, n (%)	VME, *n* (%)	ME, *n* (%)	mE, *n* (%)	UR, *n* (%)	ATU, *n* (%)	CA, *n* (%)	VME, *n* (%)	ME, *n* (%)	mE, *n* (%)	UR, *n* (%)	ATU, *n* (%)	CA, *n* (%)	VME, *n* (%)	ME, *n* (%)	mE, *n* (%)	CA, *n* (%)	VME, *n* (%)	ME, *n* (%)	mE, *n* (%)
***Enterobacterales*** ***n* = 24 isolates**	TZP *n* = 24	0 (0)	12 (50.0)	11 (91.7)	0 (0)	1 (16.7)	0 (0)	0 (0)	13 (54.2)	10 (90.9)	0 (0)	1 (20.0)	0 (0)	0 (0)	15 (62.5)	9 (100)	0 (0)	0 (0)	0 (0)	22 (91.7)	2 (33.3)	0 (0)	0 (0)
	CAZ *n* = 24	0 (0)	2 (8.3)	19 (86.4)	1 (20.0)	0 (0)	2 (9.1)	0 (0)	1 (4.2)	21 (91.3)	0 (0)	0 (0)	2 (8.7)	0 (0)	3 (12.5)	19 (90.5)	0 (0)	0 (0)	2 (9.5)	21 (87.5)	0 (0)	0 (0)	3 (12.5)
	MEM *n* = 24	0 (0)	2 (8.3)	11 (95.5)	–	0 (0)	1 (4.5)	0 (0)	0 (0)	14 (95.8)	0 (0)	0 (0)	1 (4.2)	0 (0)	0 (0)	23 (95.8)	0 (0)	0 (0)	1 (4.2)	24 (100)	0 (0)	0 (0)	0 (0)
	CIP *n* = 24	0 (0)	5 (20.8)	17 (89.5)	0 (0)	0 (0)	2 (10.5)	0 (0)	3 (12.5)	20 (95.2)	0 (0)	0 (0)	1 (4.8)	0 (0)	3 (12.5)	20 (95.2)	0 (0)	0 (0)	1 (4.8)	18 (75.0)	2 (28.6)	0 (0)	4 (16.7)
	AMK *n* = 24	0 (0)	9 (37.5)	14 (93.3)	1 (50.0)	0 (0)	0 (0)	0 (0)	6 (25.0)	17 (94.4)	1 (50.0)	0 (0)	0 (0)	0 (0)	5 (20.8)	18 (94.7)	1 (50.0)	0 (0)	0 (0)	22 (91.7)	1 (33.3)	0 (0)	1 (4.2)
	SXT *n* = 24	2 (8.3)	0 (0)	22 (100)	0 (0)	0 (0)	0 (0)	0 (0)	0 (0)	24 (100)	0 (0)	0 (0)	0 (0)	0 (0)	0 (0)	24 (100)	0 (0)	0 (0)	0 (0)	24 (100)	0 (0)	0 (0)	0 (0)
	**Total** ***n* = 124**	**2 (1.4)**	**30 (20.8)**	**104 (92.9)**	**2 (7.7)**	**1 (1.2)**	**5 (4.5)**	**0 (0)**	**23 (16.0)**	**115 (95.0)**	**1 (3.4)**	**1 (1.1)**	**4 (3.3)**	**0 (0)**	**26 (18.1)**	**113 (95.7)**	**1 (3.7)**	**0 (0)**	**4 (3.4)**	**131 (91.0)**	**5 (15.6)**	**0 (0)**	**8 (5.6)**
***P. aeruginosa*** ***n* = 4 isolates**	TZP *n* = 4	–	–	–	–	–	–	1 (25.0)	1 (25.0)	2 (100)	–	–	0 (0)	0 (0)	3 (75.0)	1 (100)	–	–	0 (0)	2 (50.0)	–	–	2 (50.0)
	CAZ *n* = 4	–	–	–	–	–	–	1 (25.0)	0 (0)	2 (66.7)	–	–	1 (33.3)	0 (0)	1 (25.0)	2 (66.7)	–	–	1 (33.3)	2 (50.0)	–	–	2 (50.0)
	MEM *n* = 4	–	–	–	–	–	–	1 (25.0)	0 (0)	3 (100)	0 (0)	0 (0)	0 (0)	0 (0)	0 (0)	3 (75.0)	0 (0)	0 (0)	1 (25.0)	4 (100)	0 (0)	0 (0)	0 (0)
	CIP *n* = 4	–	–	–	–	–	–	1 (25.0)	0 (0)	3 (100)	0 (0)	–	0 (0)	0 (0)	0 (0)	4 (100)	0 (0)	–	0 (0)	3 (75.0)	0 (0)	–	1 (25.0)
	AMK *n* = 4	–	–	–	–	–	–	1 (25.0)	0 (0)	3 (100)	0 (0)	0 (0)	0 (0)	0 (0)	0 (0)	4 (100)	0 (0)	0 (0)	0 (0)	4 (100)	0 (0)	0 (0)	0 (0)
	FEP *n* = 3	–	–	–	–	–	–	1 (33.3)	0 (0)	1 (50.0)	0 (0)	0 (0)	1 (50.0)	0 (0)	0 (0)	2 (66.7)	0 (0)	0 (0)	1 (33.3)	2 (66.7)	0 (0)	0 (0)	1 (33.3)
	**Total** ***n* = 23**	**–**	**–**	**–**	**–**	**–**	**–**	**6 (26.1)**	**1 (4.3)**	**14 (87.5)**	**0 (0)**	**0 (0)**	**2 (12.5)**	**0 (0)**	**4 (17.4)**	**16 (84.2)**	**0 (0)**	**0 (0)**	**3 (15.8)**	**17 (73.9)**	**0 (0)**	**0 (0)**	**6 (26.1)**
***A. baumannii*** ***n* = 2 isolates**	MEM *n* = 2	0 (0)	0 (0)	2 (100)	0 (0)	0 (0)	0 (0)	0 (0)	0 (0)	2 (100)	0 (0)	0 (0)	0 (0)	0 (0)	0 (0)	2 (100)	0 (0)	0 (0)	0 (0)	2 (100)	0 (0)	0 (0)	0 (0)
	CIP *n* = 2	0 (0)	0 (0)	2 (100)	0 (0)	–	0 (0)	0 (0)	0 (0)	2 (100)	0 (0)	–	0 (0)	0 (0)	0 (0)	2 (100)	0 (0)	–	0 (0)	2 (100)	0 (0)	–	0 (0)
	AMK *n* = 2	0 (0)	2 (100)	–	–	–	–	0 (0)	0 (0)	2 (100)	0 (0)	0 (0)	0 (0)	0 (0)	0 (0)	2 (100)	0 (0)	0 (0)	0 (0)	2 (100)	0 (0)	0 (0)	0 (0)
	SXT *n* = 2	0 (0)	1 (50.0)	1 (100)	–	0 (0)	0 (0)	0 (0)	0 (0)	2 (100)	0 (0)	0 (0)	0 (0)	0 (0)	0 (0)	2 (100)	0 (0)	0 (0)	0 (0)	2 (100)	0 (0)	0 (0)	0 (0)
	**Total** ***n* = 8**	**0 (0)**	**3 (37.5)**	**5 (100)**	**0 (0)**	**0 (0)**	**0 (0)**	**0 (0)**	**0 (0)**	**8 (100)**	**0 (0)**	**0 (0)**	**0 (0)**	**0 (0)**	**0 (0)**	**8 (100)**	**0 (0)**	**0 (0)**	**0 (0)**	**8 (100)**	**0 (0)**	**0 (0)**	**0 (0)**

*AMK*, amikacin; *AST*, antimicrobial susceptibility testing; *ATU*, area of technical uncertainty; *CA*, categorical agreement; *CAZ*, ceftazidime; *CIP*, ciprofloxacin; *FEP*, cefepime; *ME*, major error; *MEM*, meropenem; *mE*, minor error; *PTZ*, piperacillin-tazobactam; *SXT*, trimethoprim-sulfamethoxazole; *UR*, unreadable zones; *VME*, very major error

### *Pseudomonas aeruginosa*

Breakpoints for *P. aeruginosa* are only described after 6 and 8 h of incubation. A total of 26.1% of the inhibition zones were unreadable after 6 h of incubation. We obtained an overall CA of 87.5%, 84.2%, and 73.9% for 6 h, 8 h and DAST, respectively (Table 1). No significant difference was observed between groups. All discordant results were mE.

### *Acinetobacter baumannii*

The tested strains were both very well readable at all time points on images obtained from WASPLab. After 4 h of incubation, we obtained 3 ATU results, which were all resolved after 6 h. No discordant results were observed for all tested antimicrobials for RAST and DAST (CA = 100%) (Table 1).

### *Staphylococcus aureus*

Results of RAST and DAST for Gram-positive bacteria are displayed in Table 2. After 4 h of incubation, five strains were readable for cefoxitin, but only one strain was readable for clindamycin. No discordant results were observed for all tested antibiotics for RAST (CA = 100%). The overall CA for DAST was 95%. No significant difference was observed between groups. Both MRSA isolates were correctly categorized as cefoxitin-resistant at 6 and 8 h of incubation (unreadable zones at 4 h).

**Table 2:** Results of rapid AST and direct AST for Gram-positive bacteria, as compared to the standardized AST

		**Rapid AST, 4 h**						**Rapid AST, 6 h**						**Rapid AST, 8 h**						**Direct AST**			
		**UR, *n* (%)**	**ATU, *n* (%)**	**CA, *n* (%)**	**VME, *n* (%)**	**ME, *n* (%)**	**mE, *n* (%)**	**UR, *n* (%)**	**ATU, *n* (%)**	**CA, *n* (%)**	**VME, *n* (%)**	**ME, *n* (%)**	**mE, *n* (%)**	**UR, *n* (%)**	**ATU, *n* (%)**	**CA, *n* (%)**	**VME, *n* (%)**	**ME, *n* (%)**	**mE, *n* (%)**	**CA, *n* (%)**	**VME, *n* (%)**	**ME, *n* (%)**	**mE, *n* (%)**
***S. aureus*** ***n* = 10 isolates**	FOX *n* = 10	5 (50.0)	1 (10.0)	4 (100)	–	0 (0)	0 (0)	0 (0)	0 (0)	10 (100)	0 (0)	0 (0)	0 (0)	0 (0)	0 (0)	10 (100)	0 (0)	0 (0)	0 (0)	10 (100)	0 (0)	0 (0)	0 (0)
	CLI *n* = 10	9 (90.0)	0 (0)	1 (100)	0 (0)	–	0 (0)	1 (10.0)	2 (20.0)	7 (100)	0 (0)	0 (0)	0 (0)	0 (0)	3 (30.0)	7 (100)	0 (0)	0 (0)	0 (0)	9 (90.0)	0 (0)	0 (0)	1 (10.0)
	**Total** ***n* = 20**	**14 (70.0)**	**1 (5.0)**	**5 (100)**	**0** **(0)**	**0 (0)**	**0 (0)**	**1 (5.0)**	**2 (10.0)**	**17 (100)**	**0 (0)**	**0 (0)**	**0 (0)**	**0 (0)**	**3 (15.0)**	**17 (100)**	**0 (0)**	**0 (0)**	**0 (0)**	**19 (95.0)**	**0 (0)**	**0 (0)**	**1 (5.0)**
***Enterococcus* spp.** ***n* = 9 isolates**	AMP *n* = 9	5 (55.6)	0 (0)	4 (100)	–	0 (0)	0 (0)	1 (11.1)	0 (0)	8 (100)	0 (0)	0 (0)	0 (0)	0 (0)	0 (0)	9 (100)	0 (0)	0 (0)	0 (0)	9 (100)	0 (0)	0 (0)	0 (0)
	VAN *n* = 9	3 (33.3)	6 (66.7)	–	–	–	–	1 (11.1)	7 (77.8)	1 (100)	0 (0)	–	0 (0)	0 (0)	8 (88.9)	1 (100)	0 (0)	–	0 (0)	8 (88.9)	1 (50.0)	0 (0)	0 (0)
	**Total** ***n* = 18**	**8 (44.4)**	**6 (33.3)**	**4 (100)**	**–**	**0 (0)**	**0 (0)**	**2 (11.1)**	**7 (38.9)**	**9 (100)**	**0 (0)**	**0 (0)**	**0 (0)**	**0 (0)**	**8 (44.4)**	**10 (100)**	**0 (0)**	**0 (0)**	**0 (0)**	**17 (94.4)**	**1 (20.0)**	**0 (0)**	**0 (0)**

*AST*, antimicrobial susceptibility testing; *AMP*, ampicillin; *ATU*, area of technical uncertainty; *CA*, categorical agreement; *CLI*, clindamycin; *FOX*, cefoxitin; *ME*, major error; *mE*, minor error; *UR*, unreadable zones; *VAN*, vancomycin; *VME*, very major error

### *Enterococcus* spp.

After 4 h of incubation, all *E. faecalis* strains (*n* = 6) were readable for vancomycin, and four were readable for ampicillin. However, for *E. faecium*, 4 h of incubation revealed insufficient growth for interpretation of inhibition zones of all included isolates (*n* = 3). No discordant results were observed for all tested antibiotics for RAST (CA = 100%) (Table 2). The overall CA for DAST was 94.4%. No significant difference was observed between groups.

### Quality control

Good results were obtained for quality control testing, with > 95% within QC ranges for both QC strains, at all time points [21]. All QC values for *S. aureus* ATCC^®^ 29213™ were within the published ranges. However, after 4 h of incubation, 37.5% of *S. aureus* isolates were insufficiently grown for interpretation. After 6 and 8 h of incubation, all *S. aureus* isolates were readable. For *E. coli* ATCC^®^ 25922™, 96.5%, 97.9%, and 95.1% were within the published ranges after 4, 6, and 8 h of incubation, respectively. All *E. coli* isolates were readable after 4 h of incubation.

## Discussion

Since the publication of the EUCAST RAST method, various clinical laboratories validated RAST in routine practices [7, 14, 22]. Cherkaoui et al. investigated the use of Colibrí™ and Radian^®^ and stated the need of validation of EUCAST RAST on this automated platform [18]. To the best of our knowledge, this is the first study to report on RAST directly from BC compared to DAST and standardized AST using the fully automated WASPLab^®^ setup from Copan, including Radian^®^ in-Line Carousel and Colibrí™.

Following Clinical and Laboratory Standards Institute (CLSI) M52 guidelines [21], RAST revealed acceptable overall CA at all time points for all groups, except *P. aeruginosa*, which revealed CA of 87.5% and 84.2% after 6 and 8 h of incubation, respectively. Lower percentages of error rates (2.7–3.6% at 6 h and 1.1–1.7% at 8 h) for *P. aeruginosa* were revealed by other investigators [7, 14]. Jasuja et al., however, did reveal higher error rates with 16.7–43.3% VME, 5.9% ME, and 19.0% mE in contrast to our results revealing solely mE for *P. aeruginosa* [22]. Most observed errors for *Enterobacterales* as well were mE. However, when using EUCAST breakpoints for short incubation, mE are inevitable considering that there are no breakpoints for susceptible at increased exposure for *Enterobacterales*. We obtained 2 VME for ceftazidime and amikacin and 1 ME for piperacillin-tazobactam at 4 h, for *Enterobacterales*. ME were observed for piperacillin-tazobactam at 6 h, and only amikacin showed VME at 6 and 8 h. However, only two amikacin-resistant isolates were included. Overall, we revealed higher error rates for *Enterobacterales* compared to previous investigations publishing error rates of 0.6–4.9% [7, 14]. No errors were observed for other groups by RAST. No statistically significant differences were observed between RAST and DAST. For DAST as well, acceptable overall CA was observed for all groups but *P. aeruginosa*. EUCAST does not recommend DAST as this method is not standardized, and differences in bacterial concentration of inoculum cause changes in results, affecting therapeutic decisions [23]. Nevertheless, this method represents the method used for routine practices in our laboratory as results are obtained 1 day earlier compared to standardized AST. DAST showed VME for *Enterobacterales* (piperacillin-tazobactam, ciprofloxacin, amikacin) and *E. faecalis* (vancomycin). When screening for ESBL and CPE, all resistant strains were detected when reading inhibition zones after 8 h of incubation. However, 1 ESBL-producing *E. coli* was missed after 4 h of incubation, and 2 carbapenemases were missed after 6 h of incubation. It has been described that zone diameters for resistant strains decrease over time [14]. ESBL screening in our study was based on ceftazidime; however, ESBL-producing isolates can be missed by reliance on this antimicrobial agent solely. ESBL production should be confirmed by ordinary confirmatory and typing procedures [24].

High percentages of ATU have been described in previous studies, especially for piperacillin-tazobactam [22]. Jonasson et al. and Akerlund et al. revealed approximately 20% of ATU for *Enterobacterales* [7, 14]*.* For *E. coli*, we obtained comparable percentages of ATU (18.6–24.8%); ATU percentages for *K. pneumoniae* were lower (9.5–11.9%). We obtained high percentages of ATU for vancomycin for *Enterococcus* spp. considering that EUCAST states that only vancomycin resistance can be predicted by the RAST breakpoints. Compared to other investigators’ results, we revealed higher percentages of unreadable strains at 6 h for *P. aeruginosa*, and at 4 h for all Gram-positive strains [7, 14]. Probably indicating zone interpretation of faint growth is more difficult when using the WASPLab^®^ Webapp compared to reading by human eye.

One of the major drawbacks for routine implementation of the EUCAST RAST method is the tight reading schedule. Inhibition zones should be read at 5 min from the stated reading time, and the plates should be re-incubated within 10 min [12]. With the use of WASPLab^®^, this problem is completely resolved. The system automatically takes pictures at programmed times and re-incubates plates immediately, allowing interpretation of inhibition zones at a later time.

Follow-up studies with larger numbers of strains are necessary to confirm the observed error rates as the relatively low number of strains included in our study imply higher error rates. Especially, more amikacin-resistant *Enterobacterales* should be implemented to obtain a higher denominator for the calculation of VME. Based on our results, we advise laboratories to read inhibition zones of *Enterobacterales* at 4 h with subject to change as well as piperacillin-tazobactam and amikacin at later time points. ESBL and CPE screening is reliable at 8 h. RAST results for *P. aeruginosa* should be interpreted with caution.

In conclusion, EUCAST RAST is a very useful supplementary tool for fast AST of positive BC. Because EUCAST breakpoints for short incubation are described for a limited number of antimicrobials and bacteria only, this method does not replace standardized AST, and we believe that all RAST results should be confirmed by standardized AST. The fully automated WASPLab^®^ provides a significant advantage as pictures are made automatically implicating that we are not strictly bound to the time points for inhibition zone interpretation. Furthermore, it allows optimization of hands-on time and standardization of (pre-)analytical steps.

## Data Availability

The data that support the findings of this study are available from the corresponding author upon reasonable request.
